# Review of Platonov’s “Sports Training Periodization. General Theory and its Practical Application” – Kiev: Olympic Literature, 2013 (part two)

**DOI:** 10.1515/hukin-2015-0055

**Published:** 2015-07-10

**Authors:** Vladimir Lyakh, Kazimierz Mikołajec, Przemysław Bujas, Ryszard Litkowycz

**Affiliations:** 1 Department of Theory of Sport and Kinesiology, Academy of Physical Education in Cracow.; 2 Department of Team Sport Games, Academy of Physical Education in Katowice.

The fifth part of the reviewed monograph entitled Macrostructure of the preparation process in athletes (pp. 375–484) is probably one of the most crucial to the book. Platonov presents in detail the following topics „Bases for year round periodization in a training process” (chapter 20), “Eastern European experience of year round periodization in a training process” (chapter 21), “American and Australian experience of year round periodization in a training process” (chapter 22), “Contemporary models of year round periodization in a training process” (chapter 23) and “Direct preparation for competition” (chapter 24).

In chapter 20, analyzing available literature Platonov refers to training methods applied in elite athletes and experience of over one hundred most qualified coaches from different countries in order to indicate that the basis of year round periodization should consist of a training program which aims at developing of fundamental features to a sports discipline assuring proper adaptation of the athlete’s body to effective functioning in competitive conditions. The order and proportion of components of the training process adequate for aims at particular levels of preparation are of great importance. Lack of realization of training tasks within this area results in a significant decrease in training efficacy.

According to Platonov’s sports training periodization theory, one of the most fundamental features of effective training within the whole macrocycle is a high correlation of the applied training loads in its particular components. All decision making connected with this process shall be based on the knowledge regarding the sports level of an athlete. Platonov indicates that importance of this phenomenon increases proportionately to the time of training. Particular programs of multicycle periodization can be presented as separate mezocycles as well as a homogeneous annual macrocycle. In its first part involving mainly general conditioning and sports discipline specific exercises, only loads specific for a given sports discipline of short duration are applied. In the later phases when training involving specific sports discipline exercises is instrumental, it is crucial to prevent de-adaptation processes. Platanov highlights that authors of block periodization theory do not agree with such an approach as block periodization is based on a set of following standard training blocks of which content is designed in order to meet short-term goals (p. 378).

Discussing training periodization of an annual cycle as part of athletes’ preparation within a several years training program (s. 378–382), Platonov indicates that during first two phases (general conditioning and sports specific training) periodization does not aim directly to prepare an athlete for a competitive period (p. 378).

According to Platonov within sports specific training, mono or two-cycle periodization is applied. The most popular and frequently used model in different sports disciplines is two-cycle periodization. Besides preparation for a competitive period, the first microcycle aims at general conditioning necessary to follow a training program of a second microcycle which terminates for an athlete by competing in the most important sporting event of a given year (p. 380). In order to avoid overtraining, total volume of training loads should not exceed 50–55% of their maximum value. Furthermore, duration of a preparatory period within a macrocycle should be 1.3–1.5 times longer when compared to a competitive period.

Taking into consideration that the main aim of a training program is to prepare an athlete for the most significant competition in a given year (the Olympic Games, World Championships), the best result may be achieved following mono and two-cycle periodization (three-cycle periodization is not that frequently applied). Based on many experts’ opinions including recommendations of the American Swimming Coaches Association, Platonov states that the most effective model is monocycle periodization with some elements of two-cycle, while in the years when the Olympic Games and World Championships do not take place, elite athletes may follow three and multicycle periodization (4–7 macrocycles) (p. 381).

During the process of reaching peak performance by an athlete which may last 8–10 years, the author suggests using various types of year round periodization (mono, two or multicycle) depending on a calendar of competitive events in which the athletes are supposed to take part, age of an athlete, aims to be achieved in a particular year, a chosen preparatory strategy.

The process of reaching peak performance is characterized not only by use of different year round periodization models, but also by significant changeability of main training variables. During one year, the athletes may perform maximum 1400–1500 hours of training which amounts to 12–15 training sessions in most intensive microcycles and a total number of 600–700 training sessions. In other years, training loads may be reduced to 900–1000 hours of training which total 8–10 training sessions during microcycles of the highest level of intensity and provide a number of 350–400 training sessions per year (p. 382).

According to the Platonov’s opinion, peak performance and a gradual decrease in performance periods are characterized by the use of diverse strategies and various models, as well as a wide range of training volume applied, the use of different training methods and diversified training loads.

During these periods, we may observe a significant decrease in total training volume (20–40%) and its intensity. Platonov claims that such an approach prevents a significant development of de-adaptation processes and allows to focus on qualitative aspects of a training process. It may also be one of preventive measures applied in order to avoid overtraining.

Platonov highlights importance of the role of competitions within the year round periodization theory, characterizes their meaning and presents their most basic types. He indicates that at present we may distinguish three fundamental approaches to planning competitions. A choice of its particular variant determines the applied type of year round periodization. Considering the three approaches we may state that each of them has some strengths and weaknesses. The first type of periodization allows athletes to use competitions as a means and a method of preparation as well as control of the training process. However, too frequent participation in competitions is also related with significant fatigue, disruption of the structure of a training process, getting accustomed to competitions and their conditions. The author does not consider this approach as the most effective when the aim is to reach peak performance for the main competition.

Contrary to the first type of year round periodization, the second approach consists of minimum participation in competitions which according to Platonov is not enough especially with regard to elite athletes.

The third approach seems most rational as it allows to use strengths and minimize weaknesses of the two first types of periodization and is based on proper and individualized selection of units related to competitions.

Platonov states that in some sports disciplines one may observe a tendency to decrease the number of competitions in which athletes are supposed to take part in order to best prepare to the most important of them. The author indicates the following reasons which may significantly contribute to disruption of efficiency of a training process:

-Localization of competitions in different countries and on different continents, what requires change of time zones and induces the necessity of acclimatization;
-Participation in competitions which coincide with most crucial phases of a training process.

The last situation may negatively affect the training process and deteriorate the mental condition of athletes what finally may lead to a decrease in athletes performance and cause a time lag of adaptive changes. Consequently, it results in a decrease in the performance level and significantly increases the risk of injury (p. 386).

For this reason, in order to reach a high level of performance in athletes, it is recommended to respect a long-term preparation period as well as general and specific components of the training process. Only on such a basis it is possible to relatively quickly achieve a peak performance level which is necessary for particular competitions. Understanding of this issue will allow to avoid some discrepancies resulting from the necessity to plan a training process with the aim to reach peak performance for the main competition of the year and participation of athletes in several other competitions through the whole annual training cycle (p. 388). In practice, models of periodization aiming at reaching peak performance during the main competition of the year based on multicycle planning were created.

The first strategy aims at best performance during the main competition of the year as all the other competitions are of secondary importance and they are perceived as a form of further preparation of an athlete and control of the training process efficacy. At present, this strategy is mainly used in the training program of national teams preparing for the Olympic Games. Usually, it consists of traditional mono, two or three-cycle models of year round periodization. Following this particular strategy, 60–70% of athletes, and in some cases even 90%, reached their peak performance during the main competition of the year.

The second strategy is characteristic for most of the elite athletes nowadays who participate in different competitions during a long period (up to 10 months) and concurrently aim at their peak performance to be reached during the main competition of the year. According to Platonov, this strategy decreases effectiveness of the training process with respect to the main competition of the year and consequently, does not ensure reaching peak performance of an athlete during this particular moment. However, with a rational approach to year round periodization, it is possible to assure a high level of performance during several competitions as well as the main competition of the year, therefore, multicycle models of year round periodization including 4–7 microcycles should be applied (p. 389).

The third strategy concerns mainly such sports disciplines where the athletes take part in several competitions through a year aiming at their peak performance every single time. It is characteristic for team sports as these athletes need to be effective in 50–70 matches spanning 6–8 or even 9–11 months.

In the following parts of chapter 20, Platonov presents in detail mono, two and three-cycle models of year round periodization. He emphasizes that regardless of the structure of an annual training process model, the structure of a macrocycle consists of relatively independent elements which are, however, connected by their character, tasks order and content, i.e. periods (preparatory, competitive, transitional), phases and mesocycles. The same elements may be assigned different main aims and tasks, they may vary in their general structure and content as specific for a given sports discipline. Some discrepancies may also be noted taking into account a training program from a multiannual perspective, particular skills and abilities of an athlete as well as a calendar of competitions and tasks of an athlete during the main competition of a macrocycle (p. 391). Models of mono, two and three-cycle year round periodization are presented in Figures 1 and 2.

In the last part of chapter 20, Platonov discusses various models of multicycle periodization highlighting their effectiveness when some basic rules which significantly differentiate them from traditional mono or two-cycle year round periodization are respected. The aforementioned observation is presented in one of the Figures (p. 397) by means of a two-cycle model of periodization applied in elite freestyle wrestlers.

Finally, Platonov states that taking into consideration all positive aspects related to the possibility of frequent participation of an athlete in competitions through a year, multicycle models of periodization including two and three cycles have one important shortcoming, i.e. they do not assure the highest level of general conditioning which, as confirmed by practitioners and researchers, requires training to be carried out for 16–20 weeks (p. 397). “Shorter and less intensive training aiming at general conditioning does not provide the necessary base even if at further stages of the training process structures such as a mezocycle or 2–3 microcycles directed at the development of fundamental for a given sports discipline motor abilities are implemented” (p. 397).

Beginning in the 1950s, in the USA the two-cycle model of periodization within an annual training program was introduced. The first macrocycle (season in a short course pool; from January to the end of March or the beginning of April) was aimed at reaching peak performance for the USA Spring Championships, while the second macrocycle (season in a long course pool; from April to August) was focused on reaching best performance for the USA Summer Championships and most important international competitions. Besides some fundamental terms (a macrocycle – a season; a microcycle – a week; last phases of preparation for the main competitions – “narrowing” of a training program), main rules regarding structuring of an annual training program in the USA developed concurrently with periodization theory in the USSR (p. 413). Platonov states that in the last decades American experts significantly developed periodization theory mainly following general trends in sport (especially taking into account increasing importance of the Olympic Games), but also on the basis of closer examination of Eastern European scientific approaches and practice. It refers not only to the theoretical basis of periodization which focuses on creation of appropriate conditions necessary to reach peak performance by athletes during the main competition of the year, but also to the terminology. Following main principles of periodization theory, best American coaches of elite swimmers significantly enlarged the body of knowledge with respect to the training process focusing on such variables as technical abilities, training equipment applied on land and in the water, methodology of structuring a training program including microcycles, mesocycles, direct preparation for the main competition, etc. (p. 414).

In order to fully present approaches to the structure of an annual training cycle of most prominent American coaches, Platonov analyses such experts as Mark Schubert, Jima Sterkel, Bob Bowman, Eddi Reese, Duke Johans, Richard King. Some of the American coaches also include in their training practice a three and four-cycle model of periodization what Platonov presents analyzing work of Hines (2008) and Maglischo (2003).

Australian coaches (Sweetenham and Atkinson (2003) and others) mainly use the two-cycle model of periodizatioon, however, with regard to sprinters in swimming the three and four-cycle model of periodization is of much frequent use. Moreover, structure and content of macrocycles in sprinters is significantly different when compared to middle and long distance swimmers.

In chapter 23, Platonov presents modern models of year round periodization which include specific demands and requirements of particular sports disciplines, a multiannual approach to the training process and a calendar of competitions. Furthermore, referring to data with regard to middle-distance runners, he discusses a three-cycle model of year round periodization in track and field which is applied before the Olympic Games; structure and content of the third macrocycle directly preceding the XXIX Olympic Games; three, four, five, six and seven-cycle models of periodization used in the training process of swimmers. Moreover, Platonov analyses year round periodization in such sports disciplines as athletics, swimming, weightlifting and others where mono and two-cycle models of periodization are of greatest importance. He also notices such models do not bring any new insights into the training process of team sports where the search and preparation of specific models of periodization as well as developing central and peripheral blocks of the traditional model of periodization that includes a calendar of competitions and principles of reaching the highest level of sports performance are of crucial meaning. According to Platonov, the second approach is most effective and of greatest potential what can be noticed in modern practice of coaches (p. 460).

However, Platonov also underlines that the phenomenon of periodization has not been that widely studied in team sports when compared to sports disciplines of cyclical character or speed strength disciplines (p. 462).

In chapter 24, Platonov examines the issue of direct preparation for a competition as he proves that this term is much larger than the “period of training narrowing” or just “narrowing” which is used in Western Europe. The term of the “period of training narrowing” was for the first time defined by an Australian researcher, Forbslon Carlile (Carlile, 1963), who supported the idea of a training process of enormous volume as according to him, volume was the most influential variable when the aim was to improve sports performance in athletes. However, in order to reach peak performance during the competitions after such a training process, Carlile recommended a decrease in workloads in the precompetitive period lasting from 2 to 4 weeks, this period was referred to as the narrowing period.

Recently, several studies have been carried out which confirmed that a well planned training program applied for a few weeks before the main competition may significantly improve sports performance. In the disciplines where the results are quantitative, this improvement may oscillate between 1.5 and 4.5%. Papoti et al. (2007) indicated that an 11 day period of “narrowing” the training process improved the performance of athletes by 1.6%. In the study by Trappe et al. (2001) where the period of “narrowing” lasted 21 days the increase in performance was between 3.0 and 4.7%. In another study by Cavanaugh and Musch (1989) who applied a 4 week period of “narrowing” the training process and implemented a program of specific tests, improvement in performance oscillated between 2.0 and 3.8%, while in the study by Kenitzen (1998) it was 4%.

The reviewed monograph is one of the most important positions in the theory of sport worldwide. Not only does the author share his opinions on the year round periodization in elite athletes, discuss strengths and weaknesses of mono and multicycle models of periodization in different sports disciplines, but he also presents a fully comprehensive analysis of modern approaches to this matter indicating their pros and cons. The monograph also involves a historical approach to periodization since its very beginning i.e. when Matwejew defined its principles. Furthermore, Platonov indicates mistakes made by different scientists including Russian experts such as Issurin, Bondarczuk, Wierchoszanskij and other foreign researchers (Bompa, Hass, etc.). According to Platonov, their approach is superficial and unilateral when citing Matwejew (1964; 1977) and ignoring his more recent and modern works (1997; 1998; 1999; 2005; 2010).

Finally, we may only encourage all interested readers to get acquainted with this excellent work of the Ukrainian researcher in order to form their own opinion on the subject.

## Figures and Tables

**Figure 1:**
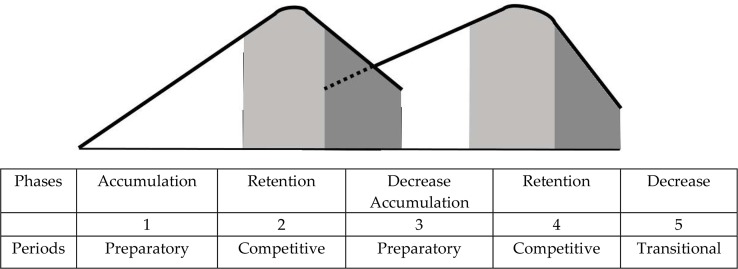
Correlation of phases of the performance level development and training periods in a two-cycle model of year round periodization (Matwejew, 1964).

**Figure 2:**
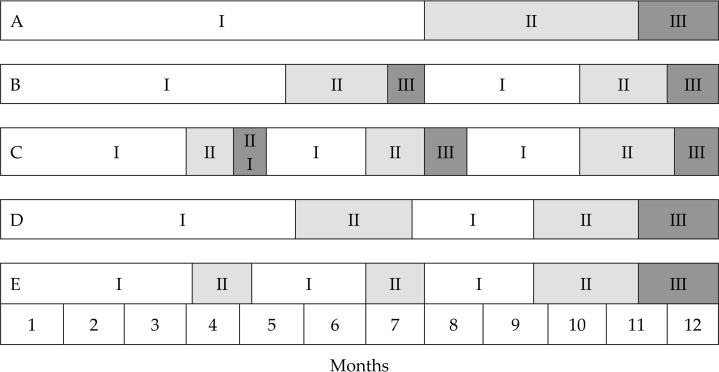
Different models of periodization within one macrocycle (12 months): A – mono-cycle planning; B – two-cycle planning; C – three-cycle planning; D – a “double” cycle; E – a „triple” cycle; I – preparatory period; I I – competitive period; III – transitional period (Platonov, 2013)
